# Counterintuitive Ballistic and Directional Liquid Transport on a Flexible Droplet Rectifier

**DOI:** 10.34133/2020/6472313

**Published:** 2020-08-19

**Authors:** Lei Wang, Jing Li, Bo Zhang, Shile Feng, Mei Zhang, Dong Wu, Yang Lu, Ji Jung Kai, Jing Liu, Zuankai Wang, Lei Jiang

**Affiliations:** ^1^Beijing Key Lab of Cryo-Biomedical Engineering and Key Lab of Cryogenics, Technical Institute of Physics and Chemistry, Chinese Academy of Sciences, Beijing 100190, China; ^2^Department of Mechanical Engineering, City University of Hong Kong, Hong Kong 999077, China; ^3^Key Laboratory of Bio-Inspired Smart Interfacial Science and Technology of Ministry of Education, School of Chemistry, Beijing Advanced Innovation Center for Biomedical Engineering, Beihang University, Beijing 100191, China; ^4^CAS Key Laboratory of Mechanical Behavior and Design of Materials, Department of Precision Machinery and Precision Instrumentation, University of Science and Technology of China, Hefei, Anhui 230027, China

## Abstract

Achieving the directional and long-range droplet transport on solid surfaces is widely preferred for many practical applications but has proven to be challenging. Particularly, directionality and transport distance of droplets on hydrophobic surfaces are mutually exclusive. Here, we report that drain fly, a ubiquitous insect maintaining nonwetting property even in very high humidity, develops a unique ballistic droplet transport mechanism to meet these demanding challenges. The drain fly serves as a flexible rectifier to allow for a directional and long-range propagation as well as self-removal of a droplet, thus suppressing unwanted liquid flooding. Further investigation reveals that this phenomenon is owing to the synergistic conjunction of multiscale roughness, structural periodicity, and flexibility, which rectifies the random and localized droplet nucleation (nanoscale and microscale) into a directed and global migration (millimeter-scale). The mechanism we have identified opens up a new approach toward the design of artificial rectifiers for broad applications.

## 1. Introduction

Billions of years' evolution has endowed many living organisms with a high level of sophistication in the directional transport of mass, momentum, and energy on their surfaces [1–4]. Directed fluid transport, adhesion, friction, and energy conversion have been widely exploited by cactus, pitcher plant, gecko, spider, lizard, and others [5–13]. Normally, the directional droplet transport observed on natural hydrophilic surfaces elegantly takes advantage of gradients in surface energy or Laplace pressure [7, 8, 12]. Over the past decade, extensive progress has been made in developing surfaces to control directional flow [14–26]. However, it remains elusive to achieve a directional and long-range liquid transport on hydrophobic surfaces [27, 28], which are particularly preferred for many applications including thermal power generation and conversion, antifogging/anti-icing, and desalination [29–40]. First, hydrophobic surfaces are associated with a limited Laplace pressure gradient or surface energy gradient. As a result, it remains difficult to achieve a long transport pathway. Second, the nonwetting properties which are typically well-preserved in benign environments can be easily lost in harsh conditions due to the complexity imposed by phase change processes [10, 11, 38, 41–46]. Thus, without proper management, the mobility of droplets is dramatically compromised due to the formation of an unwanted liquid film. To date, it remains a far prospect to fabricate new materials that endow the directed and long-range transport of liquid in a wide spectrum of working environments.

## 2. Result and Discussion

**Figure 1:**
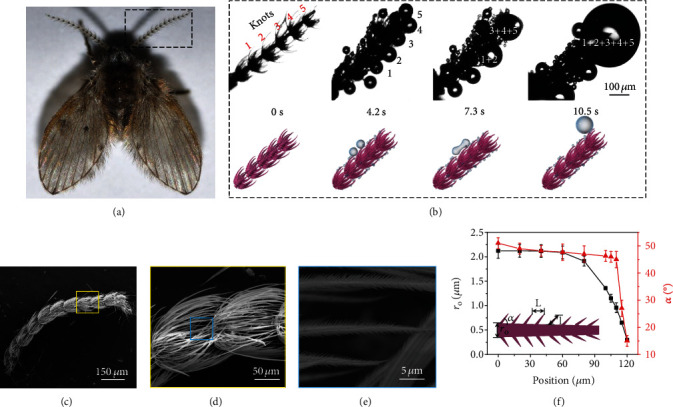
Directional and long-range droplet ballistic transport processes. (a) Optical image of drain fly. (d) Microscopic visualization and the corresponding sketches of droplet dynamics on drain fly tentacle. Initially, very small condensate droplets form randomly within individual knot on the tentacle surface and migrate unidirectionally to the apex of tentacle when they grow larger. The chain reaction of droplet transport against gravity is facilitated by frequent coalescence with neighboring droplets and is further synergized by the guided droplet relay between individual knots. (c) Scanning Electron Microscopy (SEM) image of a single tentacle of drain fly. The tentacle consists of several periodical parabola-shaped knots, with a length of ~1.5 mm. (d) The magnified view of the tentacle structure. The knot is parabola-shaped, besieged by seta arrays with a length of ~120 *μ*m. (e) The magnified side view of ratchet arrays on a single seta. The length and the center-to-center spacing of ratchets are ~1.26 *μ*m and ~0.49 *μ*m, respectively. (f) The variation of tilt angle (*α*) of ratchets (the red triangular dotted line) and the trunk diameter (*r*_o_) of the microseta (the black squared dotted line) as functions of spatial location. Here, 0 *μ*m corresponds to the bottom of seta, while 120 *μ*m corresponds to the apex. The error bars denote the standard deviation of the measurements.

Drain fly, a ubiquitous insect surviving in a very high humidity environment, develops an elegant solution to meet these demanding challenges. As shown in Figure 1(a) and Figure S1, the entire body of a drain fly maintains the high water repellency through the continuous coalescence and directional transport of condensate droplets (Figure S2).  Figure 1(b) shows the representative optical microscopy images of the condensation process on the tentacle of a drain fly, on which all the condensate droplets are efficiently transferred to the tip of the entire tentacle for final removal. Careful inspection reveals that the delicate tentacle consists of several periodical parabola-shaped knots. Initially, tiny droplet nucleates and grows within an individual knot. With time progression, the growing droplet reaches the apex of individual seta rendered by frequent coalescence with neighboring droplets. These two processes are further synergized by the guided droplet relay between individual parabola-shaped knots, after which the condensate droplets are collected to the tip of the tentacle. As a result of such a ballistic propagation, the droplet transport pathway is measured up to ~1.5 millimeters. Interestingly, such a ballistic transport behavior is in striking contrast to that on living organisms and biomimetic materials where the droplet tends to move from the apex to the base surface aided by the Laplace pressure gradient (curvature) and/or surface energy gradient [8, 47]. Finally, droplets are easily shed off via the vibration of the flexible tentacle. Despite the high humidity, we did not observe apparent liquid flooding on the tentacle surface as encountered on other conventional superhydrophobic surfaces [48]. Similar phenomena were also observed in the other parts of the drain fly (Figure S2). Thus, the drain fly can be treated as a droplet rectifier which allows a directional and ballistic propagation.

To interpret such a peculiar phenomenon, we first examined the structural morphology of the drain fly tentacle. Notably, each parabola-shaped knot is covered by tapered seta arrays (Figures 1(c) and 1(d)). The length and apex angle of the tapered seta are ~120 *μ*m and ~15°, respectively (Figure 1(e)), leading to a variation of seta radius *r*_o_ as shown in Figure 1(f) (the black square dotted line). The tips of seta arrays extend to subsequent knot, forming a seamless and continuous relay along the entire tentacle. Each seta is decorated with nanoscale ratchets (Figure 1(e)), which are inclined toward the tip of the tapered seta with a tilt angle (*α*) varying from ~51° at the bottom to ~15° at the apex (Figure 1(f), the red triangular dotted line). The length (*l*) and center-to-center spacing (*L*) of ratchets are ~1.26 *μ*m and ~0.49 *μ*m, respectively, both of which are dramatically larger than the critical droplet nucleation size predicted by the classical nucleation theory [49, 50]. Thus, during the condensation process, a liquid water phase is expected to nucleate and grow inside the nanoratchets without a spatial preference.

**Figure 2:**
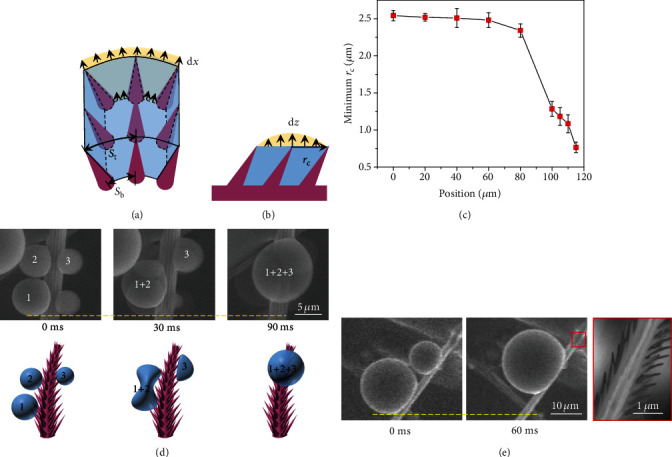
Microscopic condensation dynamics. (a) Schematic image showing the extension of liquid in the lateral direction by an incremental distance *dx*. *S*_b_ and *S*_t_ represent the space between two neighboring setae at the bottom and apex of nanoratchets. (b) Schematic image showing the extension of liquid along vertical direction by an incremental distance *dz*. *r*_c_ indicates the base area of condensate droplet. As the vapor-to-liquid phase change proceeds continuously, condensate embryo nucleating within the nanoratchet arrays extends laterally, until it grows large enough to be confined by the underlying nanostructure. (c) The minimum base area of droplet as a function of position. Here, position 0 *μ*m represents the bottom of setae. The error bars are the standard deviation of the measurements. (d) ESEM images and their corresponding schematic images representing the continuous propagation of multiple droplets in a step-by-step manner. (e) ESEM images showing the directional motion of droplet enabled by the coalescence with adjacent droplet. Remarkably, condensate droplets always move towards the tip of seta even in the case when small droplet merges with large droplet. The picture with red border demonstrates the direction of the underlying substrate.

How is the random nucleation of tiny condensate droplet inside the ratchets rectified into a directional and long-range motion spanning over several length scales? We first elucidate the initial nucleation and growth dynamics of tiny water droplets based on the interfacial energy analysis. To theoretically predict the minimum droplet base radius (*r*_**c**_) for the dewetting transition, we assume that *n* × *n* unit cells of nanoscale ratchets are first filled with water film (Figures 2(a) and 2(b)). Such a water film tends to expand either in the lateral direction, *i.e.*, along the tilt nanoscale ratchet (Figure S3 and Data file S1) or the vertical direction. When the energy cost for water film to grow in a vertical direction (Δ*E*_**nz**_) is smaller than that in the lateral directions (Δ*E*_**nx**_), or Δ*E*_**n**_^∗^ = Δ*E*_**nz**_/Δ*E*_**nx**_ < 1, a preferential growth in the vertical direction will occur, rendering droplet with enough mobility for directional transport. For an incremental volume, we have

(1) $$\begin{array}{c} \Delta E_{n}^{*}=\frac{2\left(L+s_{t}\right)}{s_{t}-\left(s_{b}-2r\right)\cos\theta_{o}-2\sqrt{{\left(l\sin\alpha \right)}^{2}+r^{2}}\cos\theta_{o}}\cdot \frac{\pi \left[{\left(r_{o}+l\sin\alpha \right)}^{2}-r_{o}^{2}\right]-Nrl\sin\alpha }{{nNLs}_{t}}<1. \end{array}$$

Here, *s*_**t**_ = 2*π*(*r*_**o**_ + *l*sin*α*)/*N* and *s*_**b**_ = 2*πr*_**o**_/*N* are the ratchet-to-ratchet spacing at the top and bottom of nanoscale ratchets, respectively. *N* is the row number of ratchet arrays decorated on a single conical seta, *r* is the radius of ratchets, and *θ*_**o**_ is the intrinsic contact angle of the drain fly surface (Data file S1). By substituting the geometric parameters into the equation (*L* = 0.49 *μ ***m**, *l* = 1.26 *μ ***m**, *r* = 0.18 *μ ***m**, *θ*_**o**_ ≈ 113^**o**^, *N* = 12, and Figure 1(f)), we can get the minimum *n*. Upon reaching this critical *n*, the condensate water film starts to inflate into the air. After that, the condensate droplet grows with base area *r*_**c**_ confined by the nanoratchets ($r_{c}=n\sqrt{s_{t}^{2}+L^{2}}/2$), until the contact angle becomes large enough for directional transport. Here, we plot the minimum *r*_**c**_ as a function of position in Figure 2(c). Clearly, the critical *r*_**c**_ for droplet transport at the top of seta is much smaller than that at the bottom of seta. Finally, the minimum radius of moving droplet can be approximately obtained as *R* ≈ *r*_**c**_/cos(*θ* − 90^**o**^), with *θ* being the average apparent contact angle, which is ~137° as evidenced by Figure 2(d). Taking the droplet sitting at the middle part of seta (region 30~60 *μ*m) as an example, the calculated minimum droplet size is 3.43 *μ*m, which is in good agreement with our experimental results shown in Figure 2(d). Notably, such a critical droplet size is ~10 times smaller than that on other natural surfaces such as butterfly wings [10].

**Figure 3:**
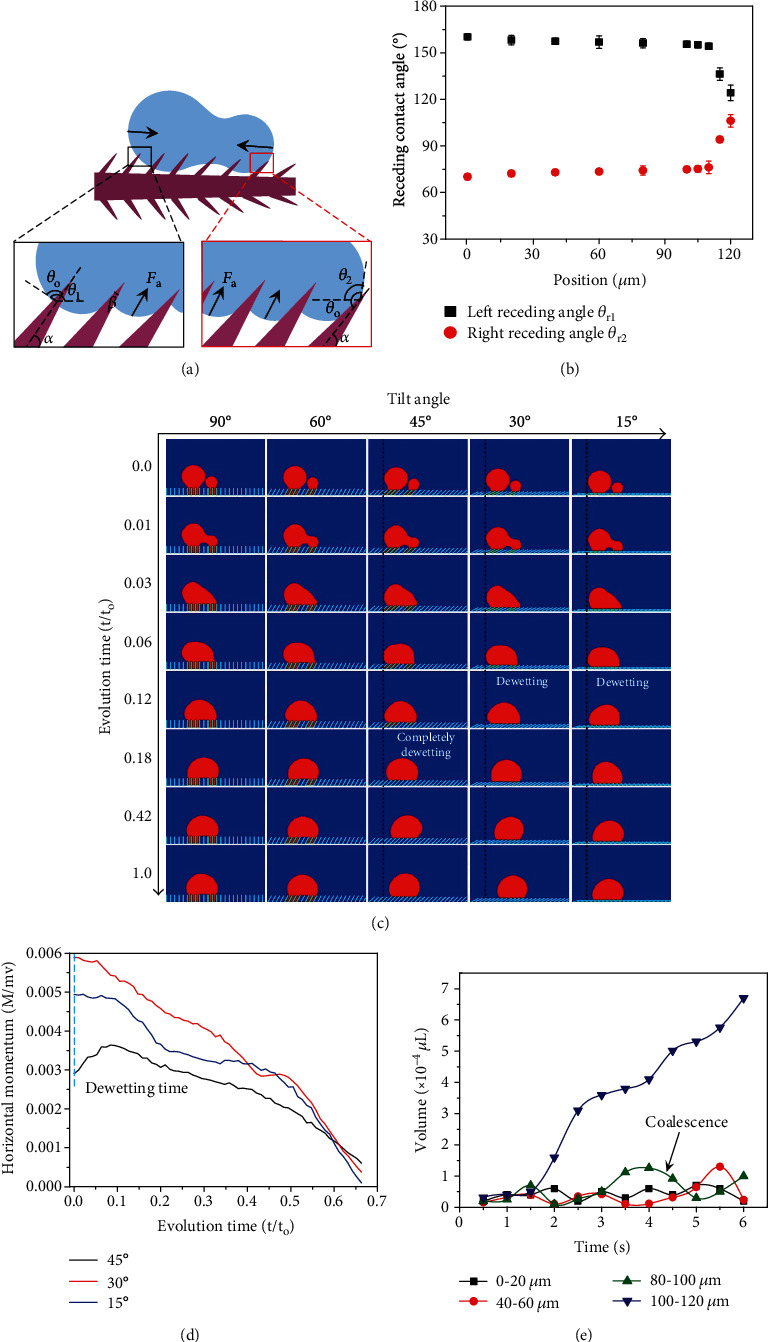
Droplet transport mechanism and characterization. (a) The sketch of coalescence process of two condensed droplets on the nanoratchets. The according receding contact angles under such unstable state can be determined by the geometric analysis in the figures with black (leftmost edge) and red (rightmost edge) borders. The unbalanced geometric induces the unidirectional coalescence of droplets. (b) The variation of calculated left and right receding angles as a function of location. Along the whole seta, the left receding contact angle is always larger than the right receding contact angle, leading to *dF*_**d**1_ > *dF*_**d**2_. The error bars represent the standard deviation of the measurements. (c) Lattice Boltzmann (LB) simulation of the coalescence process of two Wenzel droplets of different size on surfaces of equivalent length and various tilt angles ranging from 90° to 15°. There is a preferential dewetting on ratchet arrays with tilt angles of 90° and 60°, respectively. For ratchet arrays with smaller tilt angles, the merging droplet could dewet from the cavities of ratchets and then move along the tilt ratchet structure, which is in contrast to the uniform surface on which the small droplet will move toward the large droplet during the coalescence. (d) Time-dependent variation of the horizontal momentum of the dewetting droplets on surfaces with a tilt angle of 15°, 30°, and 45°, respectively. The time 0 is counted when the droplet completely dewets from the ratchets (in the vertical direction). *M* means the momentum, *m* is the mass, and *v* represent the velocity in lattice unit. *t* and *t*_o_ are the evolution time and total evolution time, respectively. (e) The variation of the volume of condensate droplets as a function of time and spatial location, with 0 *μ*m and 120 *μ*m being the bottom and apex of seta, respectively.

After growing large enough, the condensate droplets migrate towards the apex of seta by coalescing with those sitting at the top (Figures 2(d) and Movie S1). Counterintuitively, such a directional transport is robust regardless of the relative size of coalescing droplets (Figure 2(e)), which is distinct from conventional surfaces such as butterfly wings where a smaller droplet is always absorbed to the larger one (Figure S4). The efficient droplet transport is ascribed to the decoration of tilt nanoratchets. As shown in Figure 3(a), during the retraction stage of the coalescence process, the driving forces at the front (*F*_**d**1_) and rear edges (*F*_**d**2_) per unit length are expressed as  *dF*_**d**1_ = *γ*_**lv**_(cos*θ*_**t**_ − cos*θ*_**r**1_)*ds* and  *dF*_**d**2_ = *γ*_**lv**_(cos*θ*_**t**_ − cos*θ*_**r**2_)*ds*, respectively. Here, *θ*_**t**_ is the apparent contact angle of water droplet during the retraction stage of droplet coalescence; *θ*_**r**1_ = *θ*_o_ + *α*_1_ − *β*/2 and *θ*_**r**2_ = *θ*_o_ − *α*_2_ + *β*/2 are the receding contact angles at the leftmost and rightmost contact lines (Figure 3(a)), with *β* being the apex angle of ratchets, *α*_1_ and *α*_2_ being the tilt angle of ratchets, and *ds* being the integrating variable along the triple-phase contact line.  Figure 3(b) plots the spatial variation of *θ*_**r**1_ and *θ*_**r**2_ along the setae from the bottom region to the apex. Clearly, based on the above equations, the unbalanced driving force is closely related to the tilt angle *α* of ratchets; thus, the presence of ratchet structure leads to a wetting asymmetry and an unbalanced force towards the apex.

To gain more insights of the effect of *α* on droplet dynamics, we further conduct two-dimensional Lattice Boltzmann (LB, Data file S2) simulation, revealing the time-evolution transport process of two droplets on ratchets of equivalent length and different tilt angles, *i.e.*, 90°, 60°, 45°, 30°, and 15°, respectively. Herein, two Wenzel droplets in the nonequilibrium state with a radius of 60 and 30 (lattice unit) are located on ratchet arrays, with the distance between two droplets (*D*/Δ*x* = 5) small enough to ensure the occurrence of droplet coalescence. During the coalescence process, a capillary bridge connecting these two droplets is formed and the triple-phase contact line of droplets is pinned by the solid structure. Thus, the movement of the droplet in the vertical direction is enabled by the competition between the release of additional surface energy and the adhesion work, which is closely dependent on the tilt angle of ratchet arrays. Indeed, according to our simulations, the manifestation of dewetting of a coalescing droplet requires that the tilt angle of the ratchet arrays should be small enough to overcome the adhesion work (Figure 3(c) and Data file S2). The dewetting process takes place on the surfaces with a tilt angle of 45°, 30°, and 15°, whereas droplets on surfaces with a tilt angle of 90° and 60° keep a Wenzel state. After the dewetting transition, the coalescing droplet displays an asymmetric contact line and moves along the tilt direction, which is in contrast to that on a uniform surface where the smaller droplet will move toward the large one after coalescence due to the Laplace pressure contrast generated between these two droplets. To further demonstrate how the presence of ratchet structures with a proper tilt angle facilitates the lateral transport, we calculated the momentum of a dewetting droplet in the horizontal direction on different ratchet arrays as a function of time (Figure 3(d)). It is clear that the dewetting droplet on ratchets with tilt angles of 45°, 30°, and 15° is always associated with positive momentum in the lateral direction, with the largest momentum occurring on ratchets with a tilt angle of ~30°. The preferred dewetting as well as counterintuitive directional transport on surfaces with a small tilt angle is consistent with our experimental observation that the tilt angle of nanoscale ratchets on a drain fly ranges between ~45° and 15°.

Note that the flexible nature of ratchet structures on the tapered seta arrays (Figure S5) may also contribute to the directionality of a moving droplet. During the transient coalescence process, the kinetic energy of the rightmost contact line can be stored inside the deformed ratchets while the rear end of the contact line is highly mobile. As a result, the condensate droplet migrates to the apex in a step-by-step manner.

To quantify the unidirectional droplet transport within the single seta, we measured the variation of droplet volume as a function of time and spatial location.  Figure 3(e) shows the volumes of a condensate droplet as a function of time and position. Notably, the liquid volume at the apex region (90~120 *μ*m) increases continuously with the time progression, which is induced by the nucleation of the droplet from the air (corresponding to the smaller slope in the volume-time curve) as well as the coalescence as evidenced by the larger volume growth rate. By contrast, the volumes of droplets at the lower regions go through both the rise and fall process, in which the reduction in the volume is due to the uphill transport as well as the absorption by droplets at the upper region of seta. It should be noted that without the coating of wax on the surface of seta, condensate droplets on a single seta migrate downhill to its root, as revealed by our control experiment (Figure S6).

**Figure 4:**
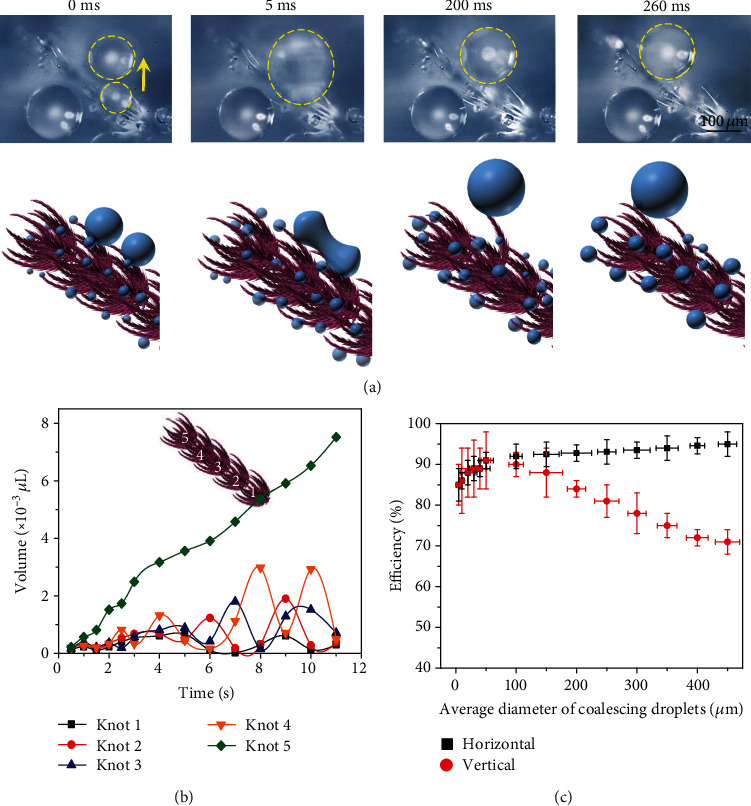
Ballistic droplet relay across knots. (a) The selected snapshots and the relative schematic images showing the ballistic transport of large droplet. Specifically, condensate droplet sitting on the tip of seta migrates over a few knots in sequence by continuous coalescence with adjacent droplets in the neighboring knot until it reaches the apex of tentacle. (b) The variation of the volumes of condensate droplets at the apex of knots as a function of time and spatial location. The maximum volumes of droplets increase gradually from knot 1 at lower region to knot 5 at apex region, suggesting the long-range transport of droplets. (c) The variation of the directional droplet transport efficiency towards the apex of tentacle as a function of average diameter of coalescing droplets when sample is horizontally (black dotted line) or vertically mounted (red dotted line). Clearly, drain fly tentacle can always maintain the directional droplet rectification even in the case of antigravity. The error bars denote the standard deviation of the measurements.

More remarkably, the ballistic droplet transport spans over the entire tentacle, with the distance three orders of magnitude larger than that of the average diameter of the tapered seta or the center-to-center spacing of ratchets. As shown in Figure 4(a) and Movie S2, the condensate droplet displays a directional migration toward the apex of knot via frequent coalescence. Upon reaching the apex, the condensate droplet merges with others sitting on a neighboring knot and continues to transport along the tentacle driven by the asymmetric contract line dynamics. Note that owing to the tapered nature of the seta, the apex of each knot is characterized with minimum solid/liquid contact area, thus promoting the facile droplet relay between knots. As a result, a large droplet can sweep the entire tentacle aided by the continuous coalescence with small droplets nearby (Figure S7). In addition, the flexibility nature of the seta can also contribute to the propagation of the droplet over knots. As shown in Figure S8, the droplet sitting between the microsetae can be squeezed out of the flexible seta array, so that the condensate droplet achieves a robust transport towards the apex of the entire tentacle.

To further characterize such a ballistic motion, we measured the volume variation of droplets at the tip of each knot as a function of time and spatial location. As shown in Figure 4(b), the volumes of droplets at knots 1 to 4 fluctuate periodically, which is a signature of droplet coalescence and refreshment. Moreover, the maximum droplet volumes increase gradually from knot 1 to knot 4, suggesting the continuous droplet relay between individual knots. Note that the volumes of droplets presented in Figure 4(b) are much larger than that on a single seta, because of the droplet coalescence between multisetae. Finally, the condensate droplets are collected to the top of the tentacle, which corresponds to the continuous increase of droplet volume of knot 5 (the top knot). These large droplets can be then shed away easily from the tapered seta arrays under external vibration, and new condensation cycle restarts.

We also compared the unique directional and ballistic droplet transport on the drain fly with other natural rectifiers. We defined the directional transport efficiency as the number of droplets directionally transported along the tilt structure relative to the number of coalescing droplets. As shown in Figure 4(c), for tiny droplets (<10 *μ*m), the directional transport efficiency is ~80%. Owing to the elegant conjunction of multiscale roughness, structural periodicity, and flexibility, such a directional migration of condensate droplets on the drain fly tentacle is still robust even in the case of antigravity. In contrast, on other natural rectifier such as *Morpho deidamia* butterfly wing [10, 51] (Figure S9 (a)), the directional transport only occurs when the droplet size is larger than a single scale (~100 *μ*m). Moreover, the directional transport easily breaks down when the surface is mounted vertically (Figures S9 (b) and (c)).

## 3. Discussion

**Figure 5:**
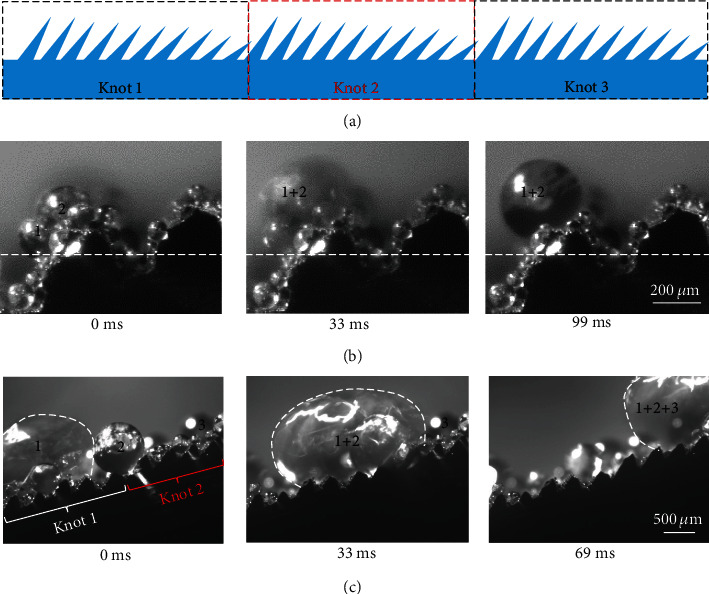
The antigravity motion of condensed droplets on the biotentacle. (a) Schematic images showing the biotentacle, which consists of periodically tilted ratchet arrays with constant spacing (*L* ~0.5 mm) and ratchet length (*l* ~0.5 mm), but varying tilt angle. The tilt angle of ratchet array in a single knot varies from 50° to 30° with a step decrease of 2°. (b) The optical image showing the coalescence of small droplets on a single ratchet, leading to a directional transport of droplets toward the top of ratchet. (c) Selected optical image showing the transport of droplet across several periodical knots. Note that although there exists a steep increase in the tilt angle between the junctions of two knots (from 30° to 50°), the droplet is still associated with a driving force toward the tilted ratchet direction, and as a result, the directional and against-gravity transport can be sustained across the entire biotentacle.

To demonstrate the generality of the peculiar ballistic droplet transport, we further designed a scaled-up flexible droplet rectifier consisting of several periodical knots using machining and soft lithography (Figure S10 and Figure 5(a)). Each knot contains microratchet arrays with uniform spacing (*L* ~0.5 mm) but varying tilt angle *α* ranging from 50° to 30°. The length of each knot is ~5 mm, which is around 50 times longer than that of the drain fly. To lower the adhesion of water on the surface, the ratchet arrays are covered by ZnO nanorods and FAS-17 (Method). As shown in Figures 5(b), the nucleated droplet can migrate uphill along a single ratchet and transport over a single knot following the tilt angle gradient direction, even against the gravity (Movie S3). Note that although there exists a steep increase in the tilt angle between the junctions of two knots (from 30° to 50°), the droplet is still associated with a driving force toward the tilted ratchet direction, and as a result, the against-gravity ballistic transport can be sustained across the entire liquid rectifier.

The sophisticated transport of the droplet in a directional and long-range fashion developed by a drain fly naturally involves an elegant conjunction of multiscale topography, structural periodicity, and flexibility and offers the potential to resolve the notorious liquid flooding imposed by the extreme environments. Our work also opens up a new principle towards the rational development of artificial liquid rectifiers to control directional transport of mass, momentum, and energy for numerous applications ranging from water harvesting and preventing ice formation to drag reduction and dropwise condensation.

## 4. Materials and Methods

### 4.1. Optical Visualization

The experiments were conducted in a customized chamber at room temperature ~25°C, which consists of an ultrasonic humidifier (SC-4317, Beijing Yadu Science and Technology Co.), cooler, and viewing window. During the measurement, the humidity was controlled at ~95 ± 5% by regulating the flow rate of a humidifier. All the samples were taped to a cooler, and the corresponding temperatures of sample surfaces were real-time monitored using K-type thermocouples (CHAL-003-BW, OMEGA). During the measurement, the surface temperature of the drain fly tentacle, scaled-up microscale ratchet, and butterfly wing were kept at ~10 ± 3°C, ~9 ± 1.5°C, and 9 ± 1.2°C, respectively. The condensation dynamics were measured using a high-speed camera (Phantom v9.1) under a frame rate of 1000 fps.

### 4.2. ESEM Visualization

All the Environmental Scanning Electron Microscopy (ESEM) images were obtained using Quanta FEG 250, FEI. During the experiment, the beam voltage was set at 15 kV, the chamber pressure was 690 Pa, and the drain fly tentacle was fixed to a Peltier cooling stage with a temperature set at ~2°C. When the relative humidity reached 100%, the chamber pressure was slowly increased from 690 Pa to 720 Pa and stabilized at 720 Pa during imaging. In this process, the condensed droplets formed and suspended on the sample surface. The droplet motion process was recorded by ESEM under a frame rate of 34 fps.

### 4.3. Energy Analysis

We conducted the interfacial energy analysis to determine the wetting state of the condensate droplet. At the initial stage of the condensation process, the droplet nucleates within the cavities of ratchets. For a water bridge occupying *n* × *n* unit cells, the surface energy cost for it to expand in a vertical direction by an incremental distance *dz* can be expressed by Δ*E*_zn_ = 2*n*(*L* + *s*_**t**_)*γ*_**lv**_*dz*. In contrast, the energy barrier for the droplet to grow along the tilt nanoratchet direction by an incremental distance *dx* can be calculated as $\Delta E_{\text{xn}}=n\gamma_{lv}\cdot \left[s_{t}-\left(s_{b}-2r\right)\cos\theta_{o}-2\sqrt{{\left(l\sin\alpha \right)}^{2}+r^{2}}\cos\theta_{o}\right]dx.$ Combined with liquid volume conservation, which can be calculated as*n*^2^*Ls*_**t**_*dz* = *n*{*π*[(*r*_**o**_ + *l*sin*α*)^2^ − *r*_**o**_^2^]/(*N*sin*α*) − *rl*} · sin*αdx*, we can finally get the surface energy ratio between lateral and vertical directions.

### 4.4. Liquid Rectifier Fabrication

The liquid rectifier was fabricated by a two-step process. First, we used a custom-designed steel knife with an apex angle of 30° to create groove arrays with a tilt angle ranging from 50° to 30° under a step decrease of 2°. The pitch and the feeding of cutting are set at ~0.5 mm, respectively. Then, we transferred the pattern from the copper mold to the PDMS substrate using the soft photolithography process. To further decorate the nanostructure on the PDMS surface, we developed a novel process using the ZnO nanoparticles as the seeding layer through spraying. After reacting in a reactor with 100 mL growth liquid (0.32 g hexamethylenetetramine and 0.76 g Zn(NO_3_)_2_·6H_2_O mixed into 100 mL deionized water) at 100°C for 12 hours, ZnO nanorods were formed. Finally, we used Heptadeca Fluorodecyltri-propoxysilane (FAS-17) to make the as-fabricated surface superhydrophobic.

## Supplementary Materials

Supplementary 1 Figure S1: morphology characterization and condensation dynamics. Figure S2: the fog repellency of drain fly body. Figure S3: the extension of condensate embryo at the initial stage. Figure S4: droplets coalescence and transport on the butterfly wing scaled surface. Figure S5: schematic image showing the deformation of nanoratchet arrays associated with contact line retraction in the coalescence process. Figure S6: in situ visualization of the condensate droplets on pretreated hydrophilic seta, which was soaked in ethanol for 1 min to remove the wax from the surface. Figure S7: the selected snapshots and schematic drawing of the sweeping of large droplet over multisetae aided by coalescence with smaller droplets, spanning over a few knots. Figure S8: the droplet transport between knots. Figure S9: condensed droplets motion on Morpho Deidamia butterfly wing. Figure S10: design of bioinspired liquid rectifier. Data File S1: the minimum energy barrier for water film to extend in a lateral direction. Data File S2: lattice Boltzmann (LB) simulation.

Supplementary 2 Movie S1: the directional and continuous transport of condensate droplets within the single seta, demonstrating a step-by-step manner.

Supplementary 3 Movie S2: the ballistic transport of droplets over multiple knots. By coalescence with the droplets sitting on the adjacent knot, the droplet can migrate across a few knots until it reaches the tip of the entire tentacle.

Supplementary 4 Movie S3: the long-distance ballistic transport of droplets on the scaled-up artificial droplet rectifier. Such a directional propagation of a condensate droplet is robust enough even under the condition of antigravity.
