# Supplementary material for: Counterintuitive Ballistic and Directional Liquid Transport on a Flexible Droplet Rectifier
Source: Research (Wash D C). 2020 Aug 19;2020:6472313. doi: 10.34133/2020/6472313 (PMC7453356; doi:10.34133/2020/6472313)
Supplement: Supplementary 1 — Figure S1: morphology characterization and condensation dynamics. Figure S2: the fog repellency of drain fly body. Figure S3: the extension of condensate embryo at the initial stage. Figure S4: droplets coalescence and transport on the butterfly wing scaled surface. Figure S5: schematic image showing the deformation of nanoratchet arrays associated with contact line retraction in the coalescence process. Figure S6: in situ visualization of the condensate droplets on pretreated hydrophilic seta, which was soaked in ethanol for 1 min to remove the wax from the surface. Figure S7: the selected snapshots and schematic drawing of the sweeping of large droplet over multisetae aided by coalescence with smaller droplets, spanning over a few knots. Figure S8: the droplet transport between knots. Figure S9: condensed droplets motion on Morpho Deidamia butterfly wing. Figure S10: design of bioinspired liquid rectifier. Data File S1: the minimum energy barrier for water film to extend in a lateral direction. Data File S2: lattice Boltzmann (LB) simulation. [file 6472313.f1.docx]

Supporting Information:

Counterintuitive Ballistic and Directional Liquid Transport on Flexible Droplet Rectifier

Lei Wang,^1^† Jing Li,^2^† Bo Zhang,^3^ Shile Feng,^2^ Mei Zhang,^2^ Dong Wu,^4^ Yang Lu,^2^ Ji Jung Kai,^2^ Jing Liu,^1*^ Zuankai Wang,^2*^ Lei Jiang ^3^

^1^ Beijing Key Lab of Cryo-biomedical Engineering and Key Lab of Cryogenics, Technical Institute of Physics and Chemistry, Chinese Academy of Sciences, Beijing 100190, P. R. China

^2^ Department of Mechanical Engineering, City University of Hong Kong, Hong Kong 999077, China

^3^ Key Laboratory of Bio-Inspired Smart Interfacial Science and Technology of Ministry of Education, School of Chemistry, Beijing Advanced Innovation Center for Biomedical Engineering, Beihang University, Beijing 100191, P. R. China

^4^ CAS Key Laboratory of Mechanical Behavior and Design of Materials, Department of Precision Machinery and Precision Instrumentation, University of Science and Technology of China, Hefei, Anhui 230027, China

E-mail address: [jliu@mail.ipc.ac.cn](mailto:jliu@mail.ipc.ac.cn) (J. L.), [zuanwang@cityu.edu.hk](mailto:zuanwang@cityu.edu.hk) (Z.W.)


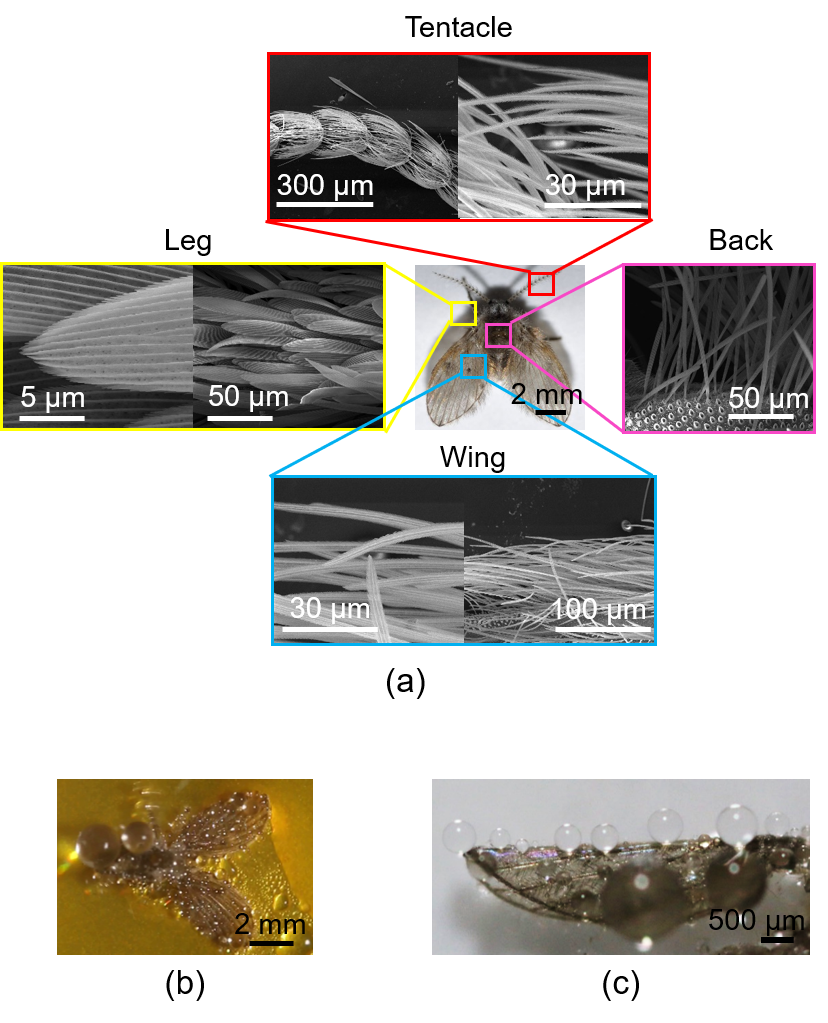


Figure S1: Morphology characterization and condensation dynamics. (a) Optical images of a drain fly and amplified SEM images showing the microstructures of the tentacle, leg, back and wing of drain fly, respectively. (b, c) Selective optical images showing the nucleation of droplets and their directional transport on the back (b) and wing (c) of drain fly, respectively.


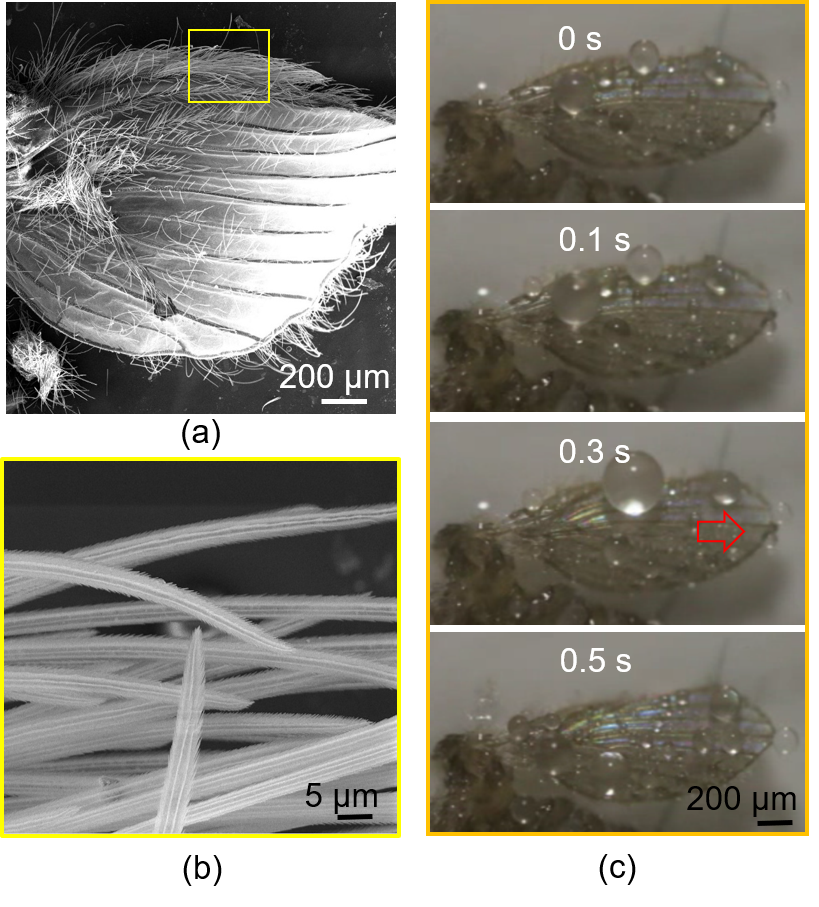


Figure S2: The fog repellency of drain fly body. (a) The SEM image of drain fly wing. (b) The magnified view of the setae decorated on the wing. (c) The directional transport and self-removal of fog along the radio direction of drain fly wing.


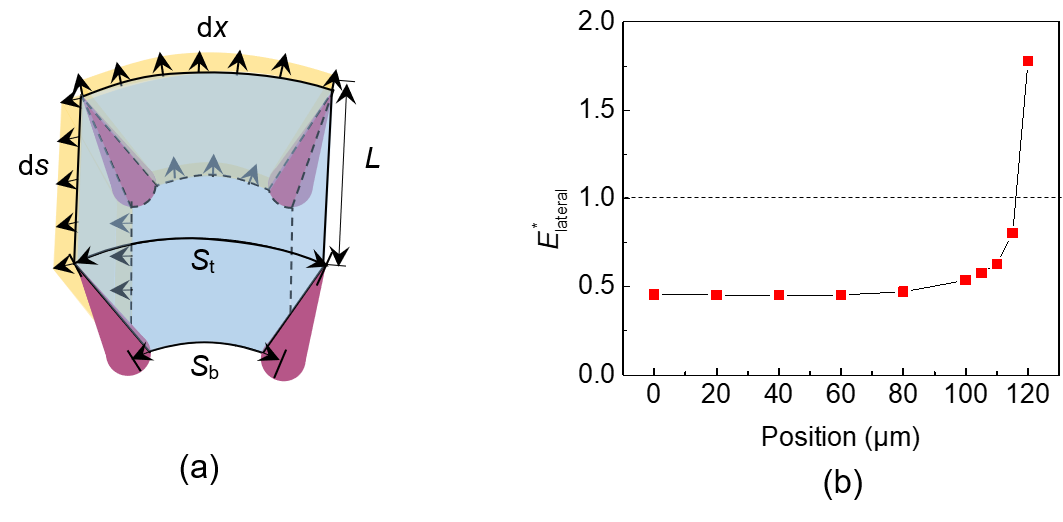


Figure S3: The extension of condensate embryo at initial stage. (a) Schematic image showing the respective energy barrier for liquid within one unit cell to propagate by an incremental distance either along tilt nanoratchet (d*x*) or across tapered seta (d*s*). *S_b_* and *S_t_* indicate the space between two neighboring setae at the bottom and apex of nanoratchets. (b) The variation of the energy barrier ratio in the lateral direction ($E_{lateral}^{*}$) as a function of position. The origin 0 μm is calculated from the bottom region of seta.


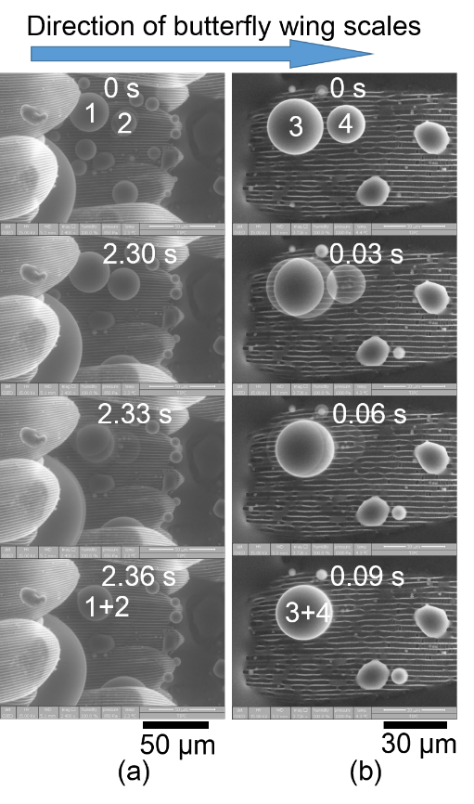


Figure S4: Droplets coalescence and transport on the butterfly wing scaled surface. The transport direction of droplets is highly affected by the uncertainty brought by the relative size of coalescing droplets.


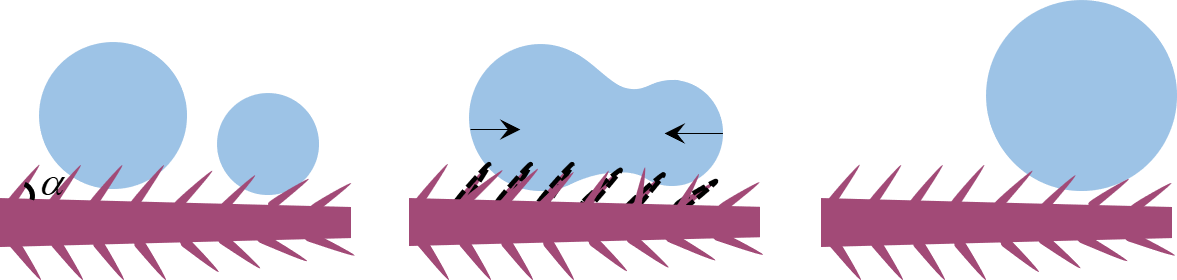


Figure S5: Schematic image showing the deformation of nanoratchet arrays associated with contact line retraction in coalescence process. The retraction of contact line reduces (increase) the tilt angle of nanoratchet ($\alpha$) at the leftmost (rightmost), which contributes to the contact line release (pinning). The dotted line represents the nanoratchets before deformation.


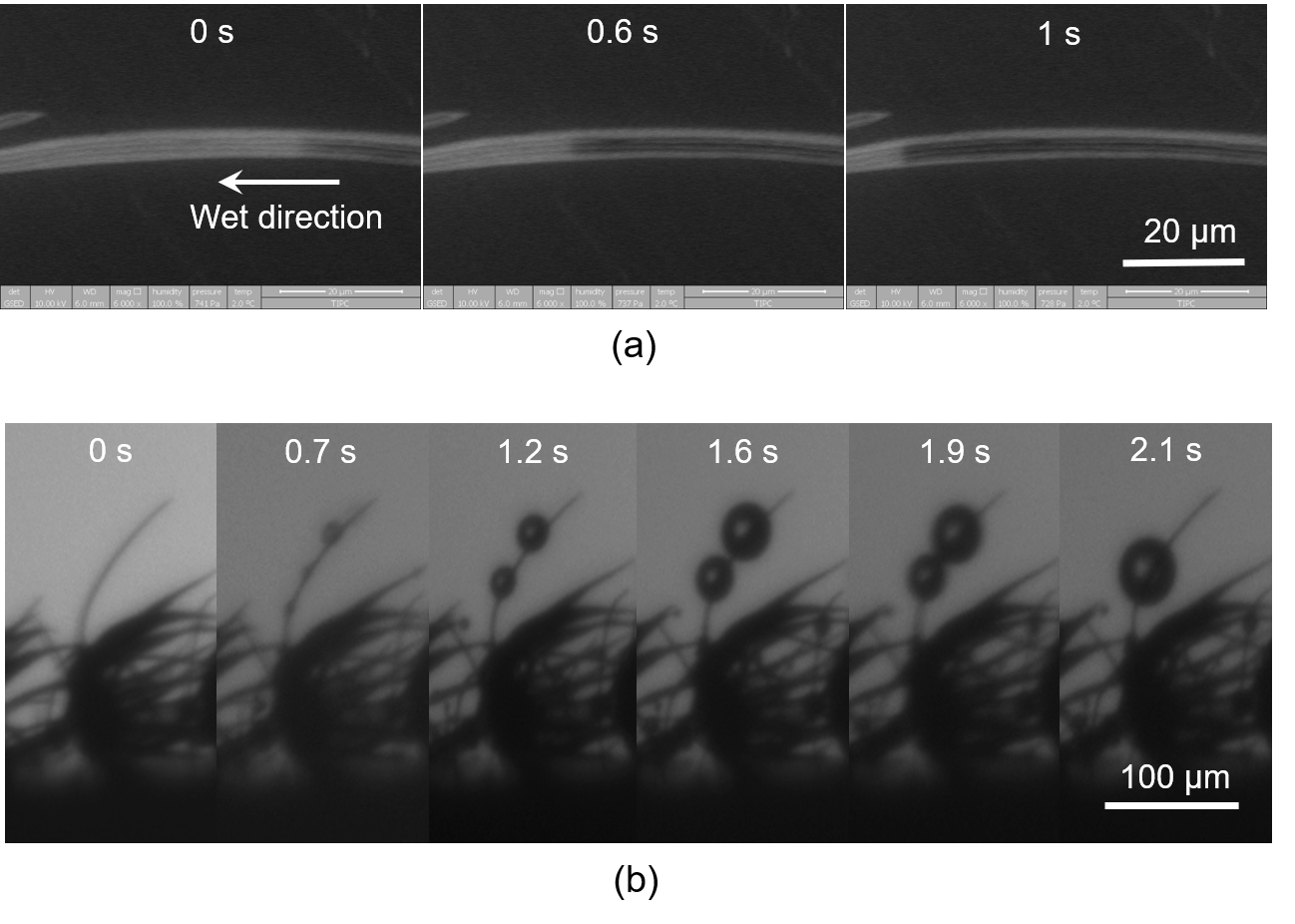


Figure S6: *In-situ* visualization of the condensate droplets on pre-treated hydrophilic seta, which was soaked in ethanol for 1 min to remove the wax from the surface. (a) The directional spreading of the initially condensed droplets along the tilt nanoratchets. As a result, a precursor and lubricant water film is formed, which contribute to the transport of larger droplets. (b) The propagation of large droplets towards the root of seta by the coalescence with each other, which is contrary to that observed on hydrophobic seta without any treatment.


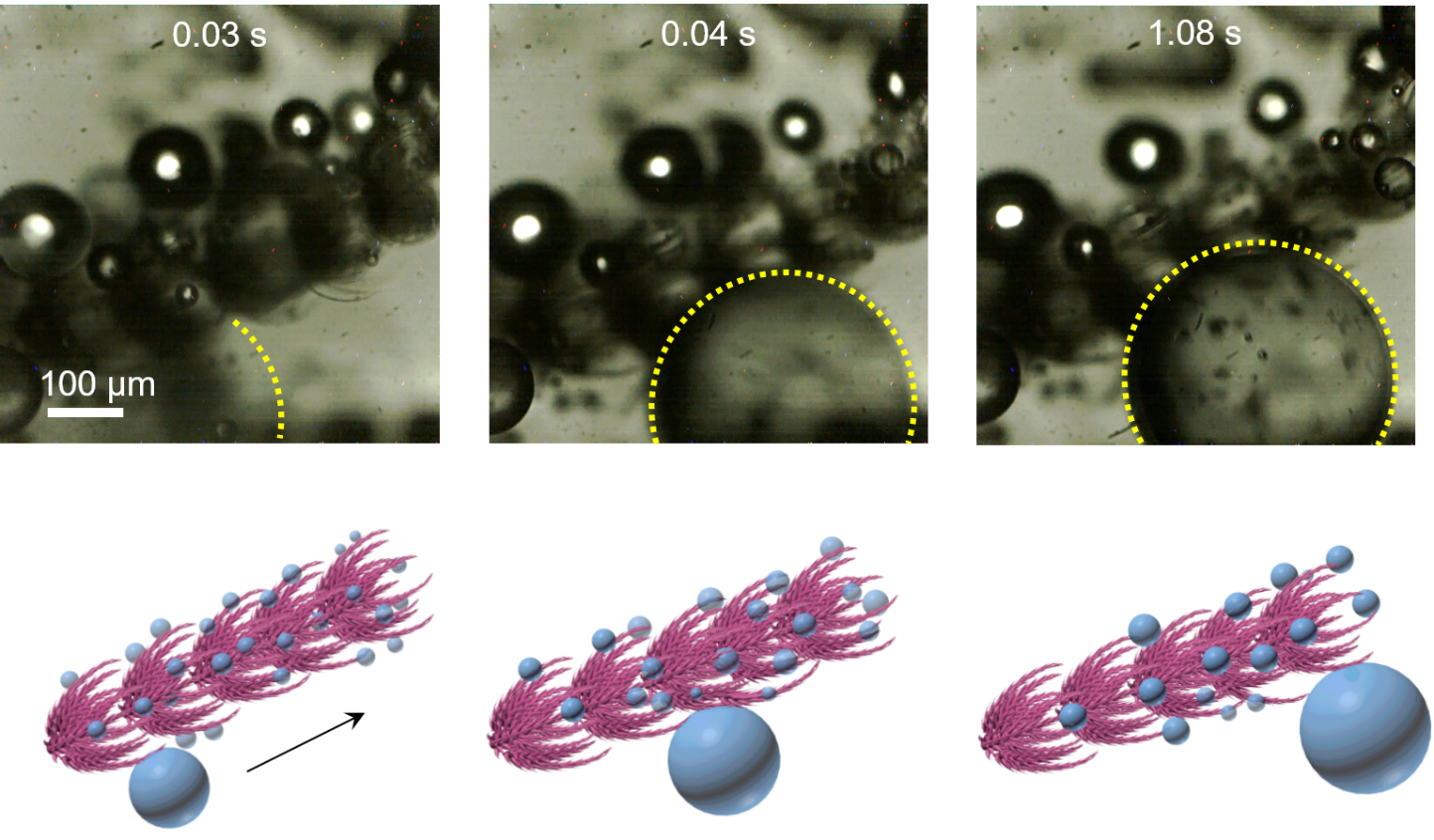


Figure S7: The selected snapshots and schematic drawing of the sweeping of large droplet over multi-setae aided by coalescence with smaller droplets, spanning over a few knots. Here, the dotted yellow line shows the outline of a large droplet which shifts over three knots.


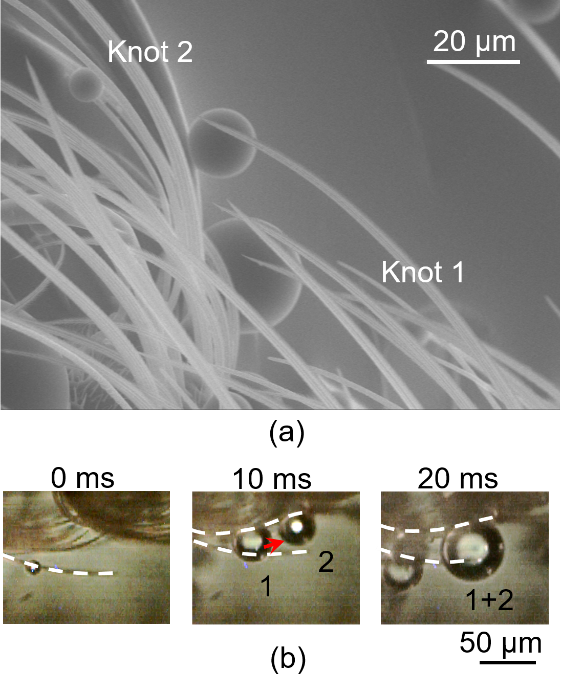


Figure S8: The droplet transport between knots. (a) ESEM images showing the droplet moving from knot 1 to knot 2. (b) The flexible ratchet guides the droplet transport to the apex of tentacle and prevents the movement in the opposite direction.


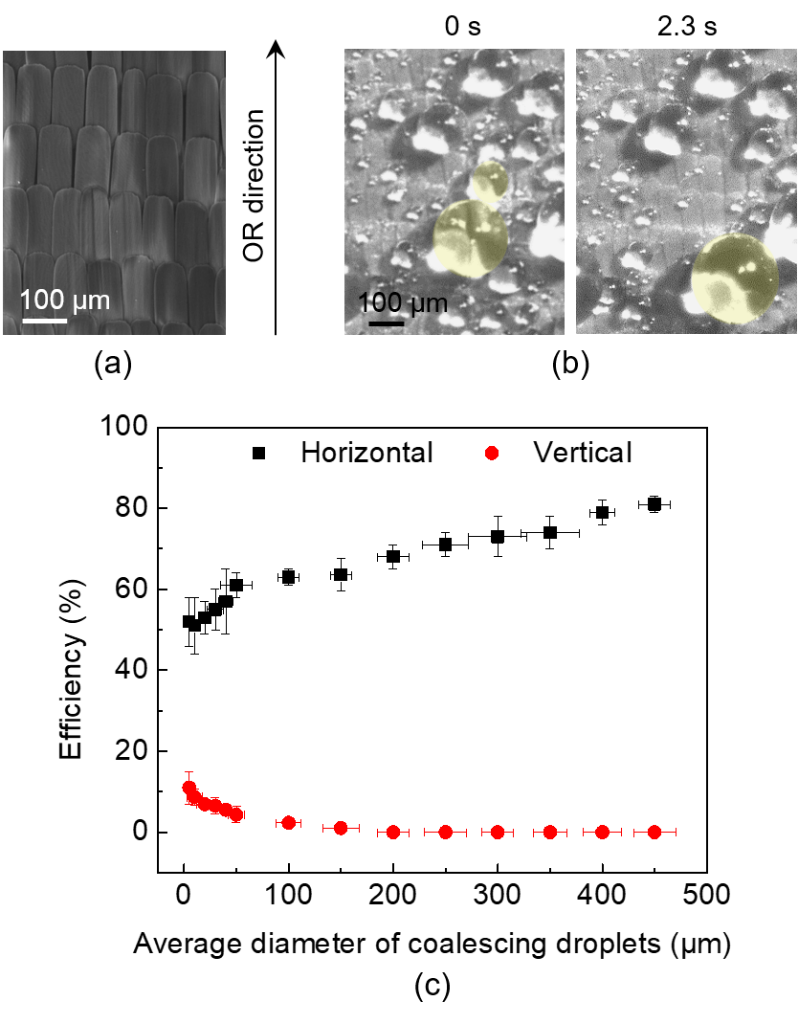


Figure S9: Condensed droplets motion on Morpho Deidamia butterfly wing. (a) SEM image showing the structure of Morpho Deidamia butterfly wing. (b) Optical images showing the droplet coalescence on vertically mounted butterfly wing. Under such an anti-gravity condition, butterfly wing easily loses its directional transport ability, and the coalescing droplets fall down along the outward radial (OR) direction. (c) The variation of the directional droplet transport efficiency along the OR direction as a function of average diameter of coalescing droplets. Condensate droplets show apparent directionality only when the surface is placed horizontally. With the increase of the droplet size, the gravity plays a dominant role and the directional droplet transfer efficiency declines. Moreover, when the sample is vertically mounted, the directional droplet transport on the butterfly wing becomes ineffective. The error bars denote the standard deviation of the measurements.


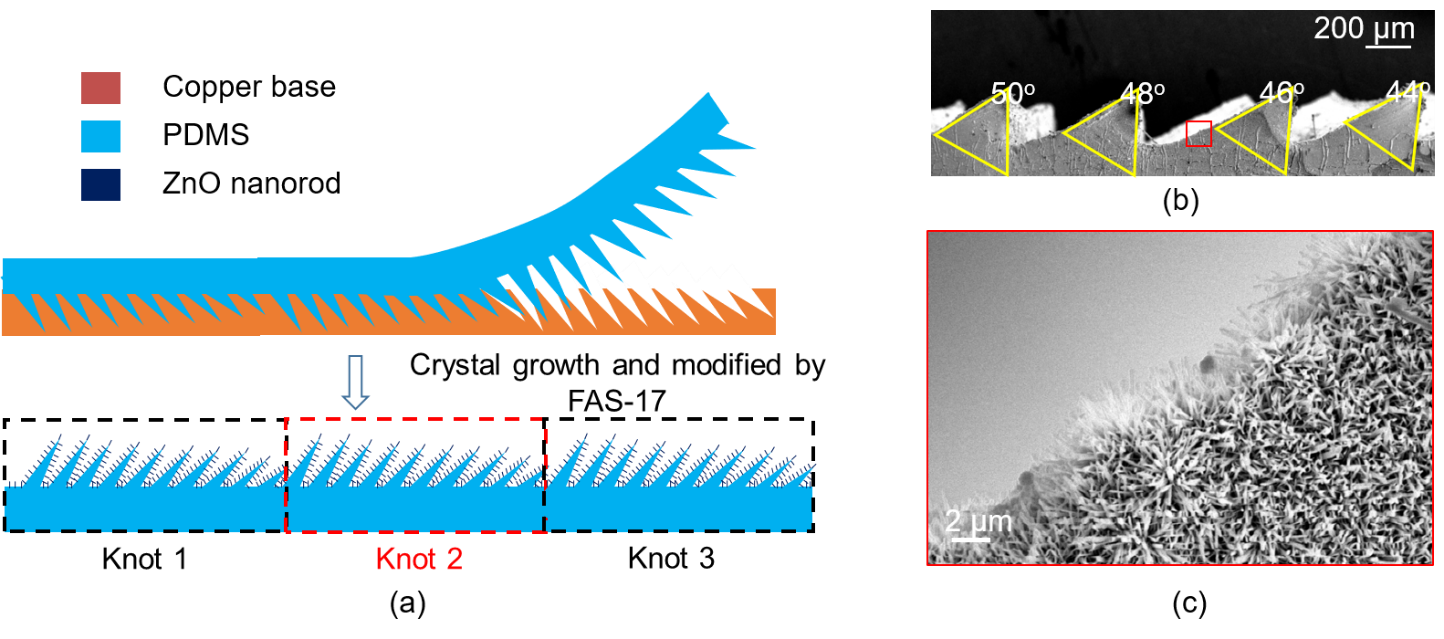


Figure S10: Design of bio-inspired liquid rectifier. We first designed a steel knife with apex angle 30^o^ to pattern the groove arrays. The tilt angles of the as-fabricated groove arrays rang from 50^o^ to 30^o^ with the tilt angle gradient of two neighboring ratchets set at 2^o^. The pitch and the feeding of cutting are controlled at ~0.5 mm, respectively. Next, PDMS is used to duplicate the topography, after which the PDMS surface is decorated with ZnO nano-rods using the enhanced crystal growth method. Details can be found in Method section.

**Data file S1.** **The minimum energy barrier for water film to extend in lateral direction.**

To elucidate the initial growth of condensate droplet in lateral direction, we consider a nucleated droplet embryo that fills in one unit cell. Owing to the structural asymmetry, the water bridge is subjected to different energy barriers in different directions. Specifically, as shown in Supplementary Fig. 2a, the surface energy cost for the condensate liquid to extend along the tilt nanoratchet by an incremental distance d*x* can be expressed as: $\Delta E_{x}=\gamma_{\text{lv}}\left[s_{t}-\left(s_{b}-2r\right)\cos\theta_{o}-2\sqrt{\left(l\sin\alpha\right)^{2}+r^{2}}\cos\theta_{o}\right]dx$, with $s_{t}=2\pi(r_{o}+$ $l\sin\alpha)/N$ and $s_{b}=2\pi r_{o}/N$, being the ratchet-to-ratchet spacing at the top and bottom of nanoscale ratchet, respectively, $\theta_{o}$ representing the intrinsic contact angle. Here, *N* is the column number of nanoratchet arrays on a single seta. In contrast, the corresponding surface energy cost across the tapered seta by an incremental distance d*s* can be approximately calculated as: $\Delta E_{s}\approx\gamma_{lv}\left[L-\left(L-2r\right)\cos\theta_{o}\right.-$ $\left.2l\cos\theta_{o}\right]ds$. By considering the same liquid volume increase, we get: $\left\{\pi\left[\left(r_{o}+l\sin\alpha\right)^{2}-r_{o}^{2}\right]/\left(N\sin\alpha\right)-rl\right\}\sin\alpha dx=l\sin\alpha\left(L-r\right)ds$. Note that here we approximate the calculation of liquid volume increase along *x* direction by neglecting the curvature of $s_{t}$ and $s_{b}$. Finally, we can obtain the energy barrier ratio for liquid to extend by an incremental volume in different directions:

$E_{lateral}^{*}=\frac{\Delta E_{x}}{\Delta E_{s}}=\frac{\left[s_{t}-\left(s_{b}-2r\right)\cos\theta_{o}-2\sqrt{\left(l\sin\alpha\right)^{2}+r^{2}}\cos\theta_{o}\right]}{L-\left(L-2r\right)\cos\theta_{o}-2l\cos\theta_{o}}\cdot\frac{N\left(L-r\right)l\sin\alpha}{\pi\left[\left(r_{o}+l\sin\alpha\right)^{2}-r_{o}^{2}\right]-Nrl\sin\alpha}$ Remarkably, the intrinsic contact angle $\theta_{o}$ can be obtained approximately using Wenzel equation. In particular, experimental result shows that the individual condensate droplet sitting at the middle part of seta (position 60μm ~ 80μm) stays in Wenzel state before coalescence, showing a stabilized average contact angle ($\theta$) of ~137^o^. Thus, $\theta_{\text{o}}=\arccos\left(\cos\theta/r_{\text{a}}\right)\approx113^{\text{o}}$, with $r_{\text{a}}\approx$ $\left\{s_{b}L-\pi r^{2}+\pi r\sin\alpha\sqrt{\left(r\sin\alpha\right)^{2}+l^{2}}\right\}/\left(s_{b}L\right)$, being the surface roughness. By substituting the geometric parameters of ratchet arrays into equation ($L=0.49\mu m$, $l=1.26\mu m$, $r=0.18\mu m$, $\theta_{o}\approx113^{o}$, $N=12$ as well as the variations of $\alpha$ and $r_{o}$ shown in Fig. 1f ), we can get the energy barrier ratio along the entire seta as a function of position (Supplementary Fig. 2b). Clearly, $E_{lateral}^{*}<1$is almost applicable to the entire seta (except for the tip), suggesting a preferential direction for liquid growth along the tilt nanoratchet.

Data file S2. **Lattice Boltzmann (LB) Simulation**

In the LB model, we hypothesize that both space and time are discrete, and particles move on a regular lattice during the consecutive propagation and collision processes. In the collision process we employed the efficient BGK model, in which the speeds of all discrete distribution functions approaching equilibrium are determined by a single relaxation time $\tau$($\tau$= 1.0 in this work). The BGK model has advantages of keeping the most fundamental conservation laws and allowing flexibility of the transport coefficients. The general form of the lattice Boltzmann equation with the BGK approximation can be written as:

$f_{i}\left(x+e_{i}\Delta t,t+\Delta t\right)=f_{i}\left(x,t\right)-\frac{\Delta t}{\tau}\left[f_{i}\left(x,t\right)-f_{i}^{eq}\left(x,t\right)\right]$ [1]

with$f_{i}\left(x,t\right)$the density distribution function,$e_{i}$ the discrete velocity, and $\Delta t$ the time step. The equilibrium distribution function, $f_{i}^{eq}\left(x,t\right)$ is given by

$f_{i}^{eq}\left(x,t\right)=\omega_{i}\rho\left[1+\frac{e_{i}\cdot u^{eq}}{c_{s}^{2}}+\frac{\left(e_{i}\cdot u^{eq}\right)^{2}}{2c_{s}^{4}}-\frac{u^{eq^{2}}}{2c_{s}^{2}}\right]$ [2]

with the weight factors $\omega_{i}=\left\{\begin{matrix} 4/9 i=0 \\ 1/9 i=1,2,3,4 \\ 1/36 i=5,6,7,8 \end{matrix}\right.$, and the speed of sound $c_{s}$=$\sqrt{3}/3$ in lattice unit. The density $\rho$is obtained as$\rho=\sum_{i} f_{i}$, the kinetic viscosity of liquid is calculated with $\upsilon=c_{s}^{2}(\tau-0.5)\Delta t$, and the macroscopic velocity is determined by

$u^{eq}=u+\frac{\tau F}{\rho}$ [3]

with $u=\frac{1}{\rho}\sum_{i=0}^{i=8} f_{i}e_{i}$. $F$contains two types of force, one is the long range interactions for liquid-liquid, liquid-vapor, vapor-vapor interactions are described using the same equation as follows:

$F_{l}\left(x,t\right)=-G\psi\left(x,t\right)\sum_{i} \omega_{i}\psi\left(x+e_{i}\Delta t,t\right)e_{i}$ [4]

where *G*­­ controls the strength of interacting and $\psi$is the interaction potential that expressed as:

$\psi(\rho)=\psi_{0}\exp(\frac{-\rho_{0}}{\rho})$ [5]

with $\psi_{0}$=4 and $\rho_{0}$= 200. The other is the interaction between solid and fluid:

$F_{s}(x,t)=-G_{s}\psi(x,t)\sum_{i} \omega_{i}s(x+e_{i}\Delta t,t)e_{i}$ [6]

G_s_ is the parameter that controls the strength of the interaction between solid and fluid sites, and $s\left(x+e_{i}\Delta t\right)$ is an indicator function that is equal to 1 or 0 for a solid or a fluid domain node, respectively. In our simulations periodic boundary condition was adopted for the right and left solid boundary, bounce back boundary condition was adopted for the top and bottom solid boundary. G was kept unchanged to fixed liquid-vapor interfacial tension. G_s_ was varied to model different intrinsic contact angle of solid surfaces. A Higher density of lattice than equilibrium state in the gas phase was set to facilitate droplet nucleation. All equations in this work were presented in lattice units, where the lattice spacing along the *x* and *y* axes and the time step were all unity ($\Delta x=\Delta y=\Delta t=1$).The lattice parameters employed in this article including the length of pillar *L/*Δ*x=*39, the spacing *S/*Δ*x=*15 and the width of pillar *W/*Δ*x=*6, which corresponding to the physical parameters of length 1.26μm, spacing 0.49μm and width of pillar 0.18μm.

To better understand the entire transport process including dewetting, coalescence and directional movement of droplets, we perform LB simulations on ratchet arrays of different tilt angles, i.e., 90^o^, 60^o^, 45^o^, 30^o^, 15^o^, respectively. Herein two Wenzel droplets with radius of 60 and 30 (lattice unit) are located on ratchet arrays, with the distance between these two droplets (*D/*Δ*x=*5) is small enough to ensure the coalescence of these two droplets in the non-equilibrium state. During the coalescence, a capillary bridge connecting these two droplets is formed and additional surface energy is converted into kinetic energy. During the coalescence process, a capillary bridge connecting these two droplets is formed and the triple-phase contact line of droplets is pinned by the solid structure. Thus the movement of droplet in the vertical direction is enabled by the completion between the release of additional surface energy and the adhesion work, which is closely dependent on the tilt angle of ratchet arrays. Indeed, our simulations reveal that the manifestation of dewetting of a coalescing droplet the ratchet arrays requires that the tilt angle should be small enough to overcome the adhesion work (Fig. 3c). Dewetting process takes place on the surfaces with tilt angle of 45^o^, 30^o^ and 15^o^, whereas droplets on surfaces with tilt angle of 90^o^ and 60^o^ keep the original Wenzel state. After the completion of dewetting, the coalescing droplet on the ratchet arrays displays an asymmetric contact line towards the ratchet direction. Generally, on a uniform surface, the coalescence droplet with a smaller radius has a larger Laplace pressure, and should move toward the bigger radius droplet as a result of Laplace pressure gradient. Thus, the counterintuitive droplet transport towards the tilted ratchet direction should result from the asymmetric morphology rendered by the ratchet structures. To demonstrate the effect of tilt ratchet arrays on the lateral transport after dewetting, we further calculate the momentum in the horizontal direction as a function of time on different ratchet arrays (Fig. 3d). The momentum of droplet can be directly obtained from the simulation formula$\rho u=\sum_{i=0}^{i=8} f_{i}e_{i}$. We find that that the droplet on surfaces with small tilt angle is always associated with positive momentum in the lateral direction (Fig. 3d). With the time evolution, the momentum decrease to zero because of the adhesion work between droplet and solid ratchet arrays. Thus a small tilt angle is favorable for the occurrence of preferred dewetting, which is consistent with our experimental observation that the tilt angle of the nanoscale ratchets on drain fly ranges between ~45^o^ and 15^o^. Taken together, these simulation results convincingly suggest that the presence of tilted ratchet structures indeed leads to an asymmetric contact line which is preferential for the lateral droplet transport.

**Movies**

Movie S1. The directional and continuous transport of condensate droplets within the single seta, demonstrating a step-by-step manner.

Movie S2. The ballistic transport of droplets over multiple knots. By coalescence with the droplets sitting on the adjacent knot, the droplet can migrate across a few knots until it reaches the tip of entire tentacle.

Movie S3. The long-distance ballistic transport of droplets on the scaled-up artificial droplet rectifier. Such a directional propagation of condensate droplet is robust enough even under the condition of anti-gravity.
